# Refractive errors in an elderly rural Japanese population: The Kumejima study

**DOI:** 10.1371/journal.pone.0207180

**Published:** 2018-11-15

**Authors:** Yoshimi Nakamura, Yuko Nakamura, Akiko Higa, Shoichi Sawaguchi, Atsuo Tomidokoro, Aiko Iwase, Makoto Araie

**Affiliations:** 1 Department of Ophthalmology, University of Ryukyu, Faculty of Medicine, Naha, Okinawa, Japan; 2 Higashi Nakano Tomidokoro Eye Clinic, Nakano, Tokyo, Japan; 3 Tajimi Iwase Eye Clinic, Tajimi, Gifu, Japan; 4 Kanto Central Hospital of the Mutual Aid Association of Public School Teachers, Setagaya, Tokyo, Japan; National Yang-Ming University Hospital, TAIWAN

## Abstract

The prevalence of refractive errors, which closely relates to visual function difficulties, several ocular disorders, and decreased quality of life, varies among countries and populations. One of the highest prevalence of myopia (spherical equivalent [SE] < -0.5 diopters [D], 41.8%) has been reported in an urban city (Tajimi) in central Japan. Here, we assess refractive conditions in a rural southwestern island (Kumejima) of Japan, where a high prevalence of glaucoma, especially angle-closure glaucoma, has been found. In Kumejima, the prevalence of myopia (SE < -0.5 D), high myopia (SE < -5 D), hyperopia (SE > +0.5 D), refractive astigmatism (cylinder > 0.5 D), and anisometropia (difference in SE between eyes > 1.0 D) were 29.5%, 1.9%, 34.1%, 38.8%, and 15.5%, respectively. Myopia decreased with age up to 70 years old but increased slightly thereafter, whereas hyperopia increased up to 70 years old and was unchanged thereafter. The prevalence of astigmatism and anisometropia was higher in older subjects. The prevalence of myopia and high myopia was higher than most of white, Hispanic, and other Asian populations, while was considerably lower than in the urban city of Japan. The high prevalence of hyperopia should be associated with high prevalence of angle closure glaucoma in this island.

## Introduction

Visual function difficulty and decreased quality of life can be caused by uncorrected refractive errors [1–3]. In addition, correction of refractive errors with spectacles, contact lenses, or refractive surgeries can be considerable economic burdens on patients and on society. Some ocular disorders are known to be associated with refractive errors. Myopia, especially high myopia, is a risk factor for macular degeneration [4], retinal detachment [5], and especially for open angle glaucoma (POAG) [6–8]. Hyperopia is a risk factor for primary angle closure glaucoma (PACG) [9] or acute ischemic optic neuropathy [10].

The prevalence of refractive errors varies among countries and populations. In population-based surveys, the prevalence of myopia (spherical equivalent [SE], < -1.0 or -0.5 diopters [D]) was 15 to 28% in Europe [11–12], the United States [13–15], and Australia [16–18], with the exception of Germany, which had a higher prevalence (35.1%) [19]. In East Asia, a prevalence of myopia exceeding 30% was reported in Indonesia [20] and Singapore [21], whereas a relatively lower prevalence (17% - 27%) was seen in India [22], Mongolia [23], Bangladesh [24], and China [25]. In Japan, one of the highest prevalence of myopia (41.8%) was reported in a population-based survey in Tajimi, a urban city in the main island [26]. More recently, an epidemiologic survey on glaucoma and other ocular conditions was conducted in Kumejima, one of the Ryukyu (formerly Okinawa) islands, which is a rural southwestern island of Japan. In Kumejima, a high prevalence of glaucoma was found [27, 28]. The aims of the current study were to evaluate the refractive errors in an elderly population in Kumejima and to compare the results with those obtained in an urban city of Honshu (the main island in Japan), Tajimi [26].

## Patients and methods

### Study population

The Kumejima study is a population-based epidemiologic survey of ocular conditions and diseases, especially of glaucoma, among all residents 40 years of age or older in Kumejima, Okinawa prefecture, Japan. This study was conducted from May 2005 through August 2006. The investigation followed the tenets of the Declaration of Helsinki and the municipal law of Kumejima Town for protecting private information. The ethics committee of Kumejima Town approved the study protocol. All participants provided written informed consent before undergoing any examinations. The number of residents 40 years or older in Kumejima was 5249 based on the registry of Kumejima inhabitants as of May 2005. All residents were invited by letter and telephone to undergo examinations at the Public Kumejima Hospital. Home visits and examinations were performed for inpatients and disabled residents. The details of the Kumejima study have been described before [27, 28] and are summarized below.

### Measurement of refractive errors

During the screening examinations, the following information was obtained: present occupation, present and past histories of ocular or systemic diseases, smoking habits, wearing contact lenses or glasses, and ocular subjective symptoms. All participants underwent measurements of subjective and objective refraction and corneal dioptric power without cycloplegia using an autokeratorefractometer (ARK-730, Nidek, Nagoya, Japan).

The best-corrected visual acuity (BCVA) was measured using Landolt rings chart at a distance of 5 m. If the uncorrected visual acuity (UCVA) was not 20/20 or worse, the refractive correction was carried out beginning with the results of autokeratorefractometry, and the corrective lenses were adjusted manually. The refractive error was measured in 0.25-D steps, and the cylindrical power was measured and recorded in the negative form. The refractive error was determined according to the results of corrective lenses that provided the BCVA in eyes in which the UCVA was worse than 20/20 and according to the results of autokeratorefractometry in eyes in which the UCVA was 20/20 or better. On slit-lamp examination, cataract was graded as 1 (mild nuclear cataract), 2 (moderate nuclear cataract), and 3 (severe nuclear cataract) with or without cortical cataract or subcapsular opacity. Measurement of the central corneal thickness using automated specular microscopy (SP-2000P, Topcon, Tokyo, Japan) also was carried out.

Emmetropia was defined as an eye with a spherical equivalent error (SE, spherical error + 0.5 x cylindrical error) between -0.5 and +0.5 D; myopia as less than -0.5, -0.75, or -1.0 D; hyperopia as more than +0.5 D; high myopia as less than -5.0 or -6.0 D. Astigmatism was determined as more than 0.5 or 1.0 D of cylinder and anisometropia as a difference of more than 1.0 D of SE between the right and left eyes. Corneal astigmatism was expressed as the difference in diopters between the steepest and flattest axes obtained by autokeratorefractometry, and it was defined as more than 0.5 D cylinder.

To assess the changes in the magnitude and the axis of astigmatism simultaneously by age, the polar value was calculated as proposed by Naeser [29] and Naeser and Hjortdal [30, 31]. Positive and negative polar values indicate with-the-rule and against-the-rule astigmatism, respectively. The difference between the refractive astigmatism and the keratometric astigmatism was determined using the vector calculation method proposed by Jaffe and Clayman [32]. In this analysis, the difference between the two forms of astigmatism was determined as the amplitude of the vector that was calculated by subtracting a vector of keratometric astigmatism from that of refractive astigmatism. Correlation of the central corneal thickness with the refractive status, including the SE, refractive astigmatism, keratometric astigmatism, and the spherical keratometric mean was also evaluated.

### Statistical analysis

All participants’ information remained private at the Data Analysis Center of Ryukyu University Faculty of Medicine. The code numbers of all participants were stored separately from all examination data in a Kumejima municipal office.

**Fig 1:**
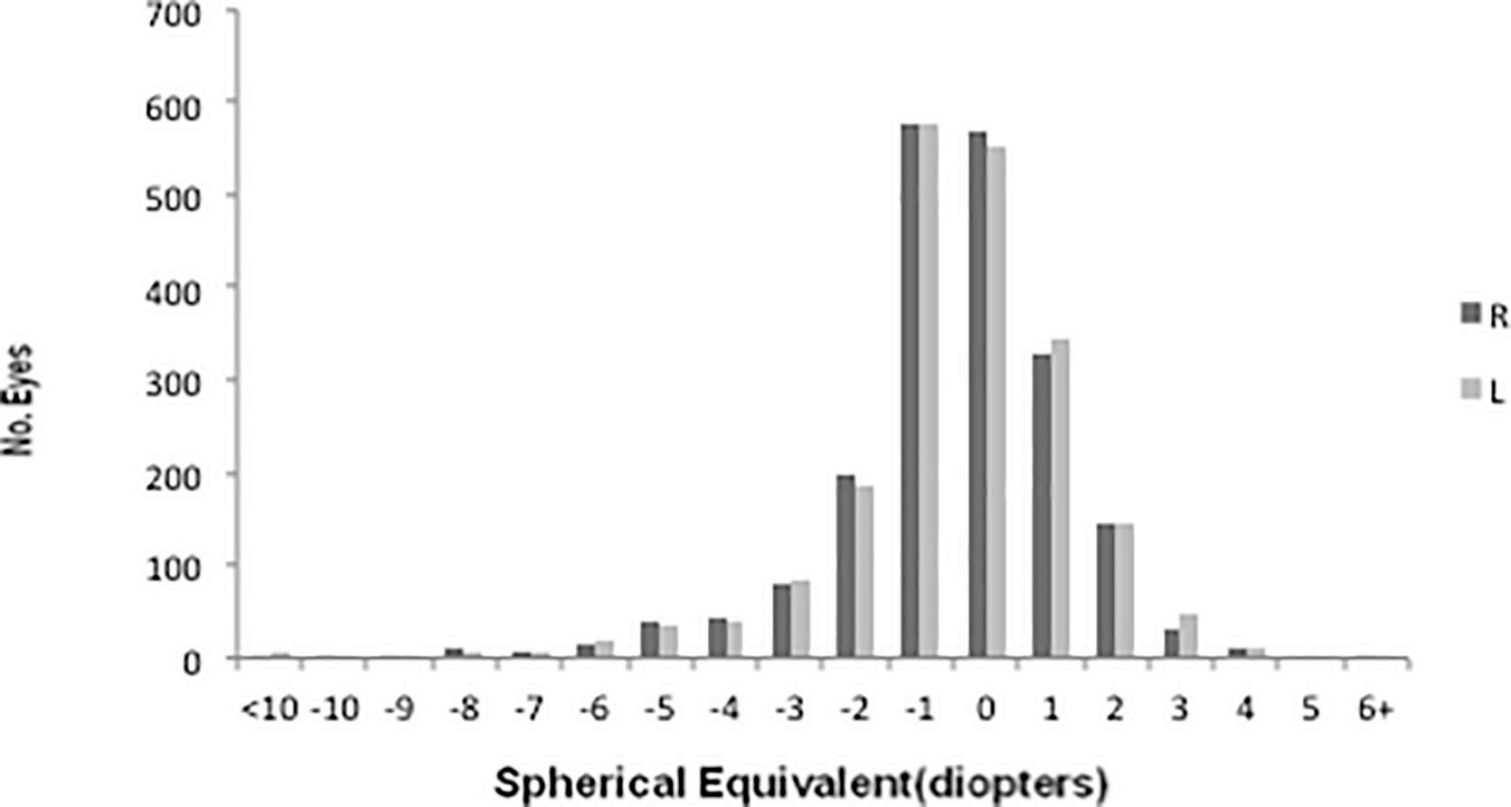
Distribution of spherical equivalent refractive errors. Bar graph showing the distribution of spherical equivalent (SE) refractive error in right and left eyes of 2067 participants for whom refractive data from both eyes were eligible. The SE refractive error in the right and left eyes averages -0.13 (95% confidence interval [CI],-0.21 to -0.05) and 0.82 (95% CI, -0.16 to 0.00), respectively.

**Fig 2:**
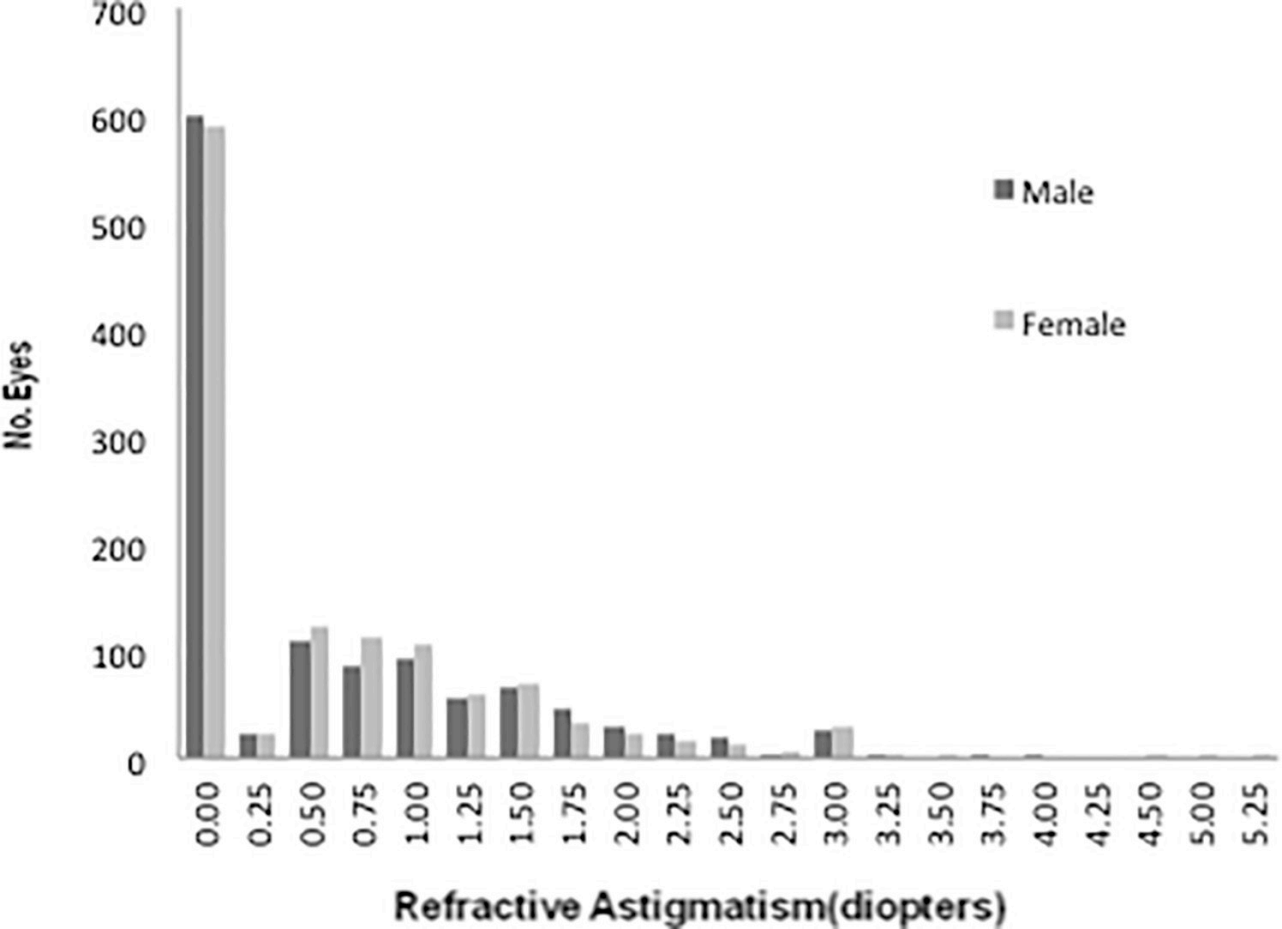
Distribution of refractive astigmatism. Bar graph showing the distribution of refractive astigmatism in 2383 right eyes of 1180 men and 1203 women. Refractive astigmatism in men and women averaged 0.62 (95% confidence interval [CI], 0.57–0.67) and 0.61 (95% CI, 0.56–0.66), respectively, without a significant intergroup difference (P = 0.41, Mann–Whitney U test).

The data were double-checked, validated through inspection, and analyzed using database software (FileMaker Pro 8; Inc., Santa Clara, CA) and statistics software (IBM SPSS Statistics version 21; IBM Japan, Tokyo, Japan) on a personal computer. Confidence intervals (CIs) were calculated with the CIA software package (BMJ Publishing Group, London, United Kingdom). Because the SE and astigmatism were not distributed normally in the current study (P < 0.05, Kolmogorov-Smirnov test, **Figs 1 and 2**), the Wilcoxon signed-rank test and the Mann-Whitney U test were used to compare the averages of the paired and unpaired samples, respectively, and the Spearman rank correlation coefficient was used for the simple correlation analysis. A P value of less than 0.05 was considered significant.

## Results

The number of registered residents 40 years of age or older in Kumejima was 5289, but 657 were identified as nonresidents, deceased, or had relocated during the screening period. Of the remaining 4632 eligible residents, 3762 (participant rate, 81.2%) completed a screening examination in the Public Kumejima Hospital (3572 participants) or in their own or nursing homes (190 participants). The participants were significantly older than the nonparticipants (61.8 ± 14.0 vs. 59.1 ± 14.9 years old, mean ± standard deviation, P < 0.001, unpaired t test,). The participant rates were similar among all age groups, except for the 90 years and older age group, and more women participated than men (P < 0.001, Fisher exact test).

Of the 7524 eyes of 3762 participants, 2730 eyes were excluded from the current analysis because reliable refractive data were not obtained (**Table 1**). Further analyses were performed on the remaining 4794 eyes. Both eyes were included in the analysis in 2068 participants, and only one eye was included in 658 participants (316 right and 342 left eyes). The mean age was significantly different among the participants for whom both eyes were included (56.9 ± 12.3 years old), those for whom only one eye was included (63.8 ± 11.9 years old), and those for whom both eyes were excluded (70.3 ±13.4 years old, P < 0.001, Kruskal-Wallis test). The sex ratios (men to women) were 1007:1060, 355:303, and 471:566 for the three groups above, respectively, with significant intergroup differences (P = 0.003, chi-square test).

**Table 1:** Reasons for exclusion from the analysis of refraction in the participants of the Kumejima study.

Reason for Exclusion	No. Eyes*	
	Right Eyes	Left Eyes
**Screened in their own or nursing homes**	192	197
**Corneal disorders**	12	19
**Pterygium**	582	599
**Aphakia**	10	13
**Pseudophakia**	421	418
**After ocular surgeries**	107	112
**Strabismus**	0	1
**Ocular trauma**	6	3
**Phthisis or prosthesis**	5	4
**Unreliable results**	17	12
**Total**	1352	1378

*These eyes were excluded from the cohort in the order shown, and no eye was duplicated. Both eyes were included in the analysis in 2067 subjects, and only 1 eye was included in 658 subjects (316 right and 342 left eyes)

The SE averaged -0.13 ± 1.85 D (95% CI, -0.21 ~ -0.05) and -0.08 ± 1.90 D (95% CI, -0.16 ~ 0.00) in the right and left eyes, respectively (Fig 1). Because the SE was highly correlated between the right and left eyes (Pearson correlation coefficient [R] = 0.88, P < 0.001,) and the averages were not significantly different (P = 0.227), only the results from the right eyes are presented.

The prevalence of refractive errors from 2383 right eyes is summarized in Table 2. The SE averaged -0.19 ± 1.61 D (95%CI, -0.29 ~ -0.10) and 0.05 ± 2.03 D (95%CI, -0.07 ~ 0.16) in men and women, respectively, with significant intergroup difference (P < 0.001). The SE was significantly correlated with age (Spearman’s rank correlation [Rs] = 0.496, P < 0.001); the prevalence of myopia decreased and that of hyperopia increased in older patients (Table 3). The prevalence of myopia (SE < -0.5 D) increased with age in participants who were more than 80 years old with eyes with significant cataract, whereas the prevalence of myopia was unchanged in eyes with no significant cataract (Table 4). The prevalence of hyperopia decreased in participants with significant cataract and appeared unchanged in those with no significant cataract except for participants who were over 80 years old. Astigmatism appeared unchanged in participants with significant cataract and increased in participants with no significant cataract.

**Table 2:** Prevalence of refractive errors in 2383 right eyes.

Refractive errors	All (N = 2383)	Men (N = 1180)	Women (N = 1203)
**Emmetropia (-0.5 to +0.5 D SE)**	36.4(34.5–38.3)	38.9(36.2–41.7)	33.9(31.3–36.6)
**Myopia (< –0.5 D SE)**	29.5(27.7–31.4)	32.0(29.4–34.7)	27.1(24.7–29.7)
**Myopia (< –0.75 D SE)**	23.7(22.1–25.5)	24.9(22.5–27.5)	22.5(20.3–25.0)
**Myopia (< –1.0 D SE)**	18.6(17.0–20.2)	19.8(17.7–22.2)	17.3(15.3–19.5)
**High myopia (< –5.0 D SE)**	1.9(1.4–2.5)	1.3(0.8–2.1)	2.5(1.8–3.5)
**High myopia (< –6.0 D SE)**	1.2(0.85–1.7)	6.8(3.4–13.3)	1.8(1.1–2.7)
**Hyperopia (> 0.5 D SE)**	34.1(32.2–36.0)	29.2(26.6–31.8)	39.0(36.3–41.8)
**Astigmatism (> 0.5 D)**	38.8(36.9–40.8)	38.3(35.6–41.1)	39.3(36.6–42.1)
**Astigmatism (>1.0 D)**	22.2(20.6–23.9)	23.1(20.8–25.6)	21.3(19.1–23.7)

D = diopters; SE = spherical equivalent. Prevalence ratios (%) are shown with 95% confidence intervals in the parentheses.

**Table 3:** The prevalence of refractive errors in 2384 right eyes in age and gender groups.

		Age group (years)				
		40–49	50–59	60–69	70–79	80+
**Emmetropia** **(-0.5 to +0.5 D SE)**	**Men**	44.0(39.1–48.9)	43.1(38.2–48.2)	30.2(24.2–36.9)	32.8(26.2–40.1)	20.9(11.4–35.2)
	**Women**	43.8(38.9–48.8)	41.6(36.1–47.2)	23.2(17.9–29.4)	21.3(16.7–26.8)	25.0(16.6–35.8)
**Myopia** **(<–0.5 D SE)**	**Men**	51.4(46.5–56.3)	29.4(25.0–34.2)	15.1(10.8–20.7)	15.8(11.1–22.0)	20.9(11.4–35.2)
	**Women**	48.6(43.6–53.6)	27.0(22.3–32.4)	7.4(4.5–11.8)	14.5(10.6–19.4)	14.5(8.3–24.1)
**High myopia** **(<–5.0 D SE)**	**Men**	2.8(1.6–5.0)	0.8(0.3–2.3)	0.0(0.0–1.9)	0.6(0.1–3.2)	0.0(0.0–0.8)
	**Women**	4.0(2.4–6.4)	4.7(2.8–7.8)	0.5(0.1–2.7)	0.0(0.0–1.5)	0.0(0.0–4.8)
**Hyperopia** **(>0.5 D SE)**	**Men**	4.6(3.0–7.2)	27.5(23.3–32.2)	54.8(47.8–61.5)	51.5(44.0–58.8)	58.1(43.3–71.6)
	**Women**	7.7(5.4–10.8)	31.4(26.4–36.9)	69.5(62.8–75.4)	64.3(58.1–70.0)	60.5(49.3–70.8)
**Astigmatism** **(>0.5 D)**	**Men**	22.1(18.3–26.5)	32.5(28.0–37.4)	50.3(43.4–57.1)	63.7(56.3–70.6)	79.1(64.8–88.6)
	**Women**	19.3(15.6–23.5)	28.4(23.5–33.8)	47.8(41.0–54.6)	65.1(59.0–70.7)	75.0(64.2–83.4)

D = diopters; SE = spherical equivalent. Prevalence ratios (%) are shown with 95% confidence intervals in the parentheses.

**Table 4:** The prevalence of refractive errors in 2384 right eyes with or without significant cataract.

		Age group (years)		
		60–69	70–79	80+
**Myopia** **(<–0.5 D SE)**	**Significant cataract**	37.5(13.7–69.4)	23.6(14.4–36.4)	30.4(19.1–44.8)
	**No significant Cataract**	10.7(8.0–14.1)	13.7(10.6–17.6)	8.2(3.8–17.0)
**High myopia** **(<–5.0 D SE)**	**Significant cataract**	12.5(2.2–47.1)	1.8(0.3–9.6)	0.0(0.0–0.8)
	**No significant Cataract**	0.0(0.0–1.0)	0.0(0.0–1.0)	0.0(0.0–5.0)
**Hyperopia** **(>0.5 D SE)**	**Significant cataract**	62.5(30.6–86.3)	50.9(38.1–63.6)	37.0(24.5–51.4)
	**No significant Cataract**	62.2(57.3–66.8)	60.3(55.2–65.2)	74.0(62.9–82.7)
**Astigmatism** **(>0.5 D)**	**Significant cataract**	75.0(40.9–92.9)	72.7(59.8–82.7)	69.6(55.2–80.9)
	**No significant Cataract**	48.5(43.6–53.4)	63.3(58.2–68.1)	80.8(70.3–88.2)

D = diopters; SE = spherical equivalent. Prevalence ratios (%) are shown with 95% confidence intervals in the parentheses.

After adjusting for age, the SE was significantly correlated with refractive astigmatism (partial correlation coefficient, PCC = 0.197, P < 0.001), spherical keratometric mean (PCC = -0.067, P = 0.001), keratometric astigmatism (PCC = -0.097, P < 0.001), and axial length (PCC = -0.343, P = 0.000), but not with central corneal thickness (PCC = -0.007, P = 0.716). Among 2068 participants for whom both eyes were eligible, the absolute differences in SE between the right and left eyes (anisometropia) averaged 0.54 ± 0.77 D (95% CI, 0.51 ~ 0.58) overall without significant difference between men (0.53 ± 0.71 D [95% CI, 0.49 ~ 0.58]) and women (0.55 ± 0.82 D [95% CI, 0.50 ~ 0.60]) (P = 0.41). Anisometropia was found in 320 (15.5%; 95% CI, 14.0% ~ 17.1%) of the 2068 participants. Anisometropia increased significantly with age (R = 0.99, P < 0.001, Table 5).

**Table 5:** The prevalence of anisometropia in age and gender groups.

Age Group (years)	Men	Women	All
**40–49**	7.0(4.8–10.2)	7.6(5.3–10.8)	7.3(5.6–9.5)
**50–59**	10.3(7.5–14.1)	9.4(6.5–13.4)	9.9(7.8–12.6)
**60–69**	20.4(14.8–27.4)	20.0(14.8–26.5)	20.2(16.2–24.8)
**70–79**	29.1(21.9–37.3)	30.3(24.3–37.0)	29.8(25.1–34.9)
**80+**	48.6(33.0–64.4)	46.4(34.0–59.3)	47.3(37.3–57.4)
**ALL**	14.5(12.5–16.8)	16.4(14.3–18.8)	15.5(14.0–17.1)

D = diopters; SE = spherical equivalent. Prevalence ratios (%) are shown with 95% confidence intervals in the parentheses.

Refractive astigmatism averaged 0.61 ± 0.80 D (95% CI, 0.58 ~ 0.65) without significant difference between men (0.62 ±0.80 D [95% CI, 0.57 ~ 0.67]) and women (0.61 ± 0.80 D [95% CI, 0.56 ~ 0.66]) (P = 0.69, Fig 2). Refractive astigmatism increased significantly with age (R = 0.376, P < 0.001), but was not correlated with the SE (R = -0.008, P = 0.692).

The spherical keratometric mean, which is the mean of the steepest and flattest meridians of the keratometric readings, averaged 44.22 ± 1.38 D (95% CI, 44.16 ~ 44.27) in 2381 right eyes and was significantly greater in women (44.52 ± 1.35 D [95% CI, 44.44 ~ 44.59]) than in men (43.91 ± 1.35 D [95% CI, 43.83 ~ 43.99], P < 0.001). The spherical keratometric mean was not correlated with age (R = 0.37, P = 0.091). Keratometric astigmatism averaged 0.90 D (95% CI, 0.87 ~ 0.92) overall and was 0.89 D (95% CI, 0.85 ~ 0.93) and 0.91 D (95% CI, 0.87 ~ 0.95) in men and women, respectively, with no significant intergroup difference (P = 0.26). Keratometric astigmatism was not correlated with age (Rs = 0.086, P < 0.001)

The polar value in keratometry decreased significantly with age (Rs = -0.30, P < 0.001). The correlation between age and the polar value was significantly (P < 0.001) stronger in refractive astigmatism than in keratometric astigmatism. The difference between refractive and keratometric astigmatism calculated with the vector method averaged 1.60 D (95% CI, 1.56 ~ 1.64) and was 1.59 D (1177 eyes) (95% CI, 1.53 ~ 1.65) and 1.61 D (95% CI, 1.54 ~ 1.67) in men and women, respectively, without significant intergroup difference (P = 0.89). The difference between refractive and keratometric astigmatism increased significantly with age (R = 0.216, P < 0.001).

The axial length was significantly correlated with age (R = -0.252, P < 0.001). After adjusting for age, axial length was shorter in women than in men (P = 0.021) and significantly associated with the SE (PCC = -0.583, P < 0.001), refractive astigmatism (PCC = 0.103, P < 0.001), spherical keratometric mean (PCC = -0.539, P < 0.001), and keratometric astigmatism (PCC = 0.013, P = 0.513). The central corneal thickness was not significantly associated with SE (R = 0.027, P = 0.19), the spherical keratometric mean (R = -0.25, P = 0.23), refractive astigmatism (R = 0.05, P < 0.001), and keratometric astigmatism (R = -0.033 P = 0.16). A significant correlation was not found between the central corneal thickness and these refractive parameters even though age was statistically adjusted (P > 0.05).

## Discussion

The current study evaluated the refractive status of people living in a rural southwest island of Japan. The study found that the crude prevalence of myopia (SE < -0.5 or -1.0 D) was 29.5% or 18.6%, respectively, which is slightly lower than in Singapore [21] (35.0% or 28.0%, respectively) and Germany [33] (35.1% or 26.2%) and is higher than that in white, Hispanic, and other Asian populations, ranging from 14% to 27% [20, 22–25, 34]. The prevalence of high myopia (SE < -5.0 or <-6.0 D) in the current study was 1.9% or 1.2%, respectively, which is again lower than that in Singapore (6.9%, SE<-5.0D) [21] and Germany (5.6%, SE < -5.0D) [33] and nearly the same as those in other Asian countries (1.7% to 3.0%) [20, 23–25, 34], and in white and Hispanic populations (1.0% to 2.4%) [12, 14, 19]. Thus, in this Japanese rural population, myopic individuals are more common than in other populations but high myopia is not necessarily as frequent.

The prevalence of hyperopia (SE > 0.5 D) in the current study was 34.1%, which is similar to other Asian countries including India (27.4%) [22], Singapore (35.9%) [21], and Mongolia (32.9%) [23]. These prevalence values of hyperopia in Asian countries are lower than those in most of white populations (47.3% - 56.6%) [14, 17] except for that in Germany (31.8%) [33]. The prevalence of anisometropia (bilateral difference in SE > 1.0 D) was 15.5% in the current study, which is similar to that in Mongolia (10.7%) [23], Bangladesh (11.9%) [24], Australia (14.1%) [17], and Germany (13.5%) [33], but is lower than that in Singapore (20%) [21], Taiwan (19.9%) [34], and Indonesia (24.3%) [20]. The rationale and clinical importance of these differences in anisometropia among countries or populations are still unclear at present.

In comparison with a previous population-based study in Tajimi, an urban city in the main island of Japan, the prevalence of myopia (SE < -1.0 D) in the current result obtained in Kumejima island was considerably lower (18.6% in Kumejima vs. 32.4% in Tajimi [26]), while that of hyperopia (SE > 0.5 D) was higher (34.1% vs. 27.9% [26]). Though the inclusion criteria of age, 40 years and older, was identical between these studies, the mean age was greater in the current study (58.4 years vs. 56.9 years [26]). However, the trend that myopia is less frequent but hyperopia more frequent in Kumejima than in Tajimi can be found in the age-stratified analysis on the prevalence of refractive errors (Table 6). To explain these differences in refractive errors between the two populations in Japan, environmental factors should be addressed. As the environmental factor, the major industries in Kumejima are agriculture and fishery in which far vision is more useful compared to working in an office. During their childhood, the participants in the current study might have played outdoors more often compared to the Tajimi participants. In previous studies, refractive status was compared between urban and rural areas. In adult populations (40 years and older), myopic refractive error was associated more with urban areas than with rural ones in China [25] and India [35]. A similar trend was also found in children in China [36], Taiwan [37], India [38], and Cambodia [39], suggesting that differences between the refractive status of adult populations in urban and rural areas should be closely associated with environmental factors during the period of growth.

**Table 6:** Comparisons of refractive errors between Kumejima and Tajimi studies.

		Emmetropia		Myopia		High myopia		Hyperopia		Astigmatism	
		(-0.5 to +0.5 D SE)		(<–0.5 D SE)		(<–5.0 D SE)		(>0.5 D SE)		(>0.5 D)	
		Kumejima	Tajimi	Kumejima	Tajimi	Kumejima	Tajimi	Kumejima	Tajimi	Kumejima	Tajimi
40–49	Men	44.0	27.6	51.4	70.3	2.8	17.7	4.6	2.1	22.1	41.1
		(39.1–48.9)	(22.8–32.4)	(46.5–56.3)	(65.4–75.2)	(1.6–5.0)	(13.6–21.8)	(3.0–7.2)	(0.6–3.6)	(18.3–26.5)	(35.9–46.4)
	Women	43.8	29.3	48.6	67.8	4.0	15.0	7.7	2.9	19.3	39.9
		(38.9–48.6)	(25–33.5)	(43.6–53.6)	(63.4–72.2)	(2.4–6.4)	(11.6–18.3)	(5.4–10.8)	(1.4–4.5)	(15.6–23.5)	(35.3–44.5)
50–59	Men	43.1	32.0	29.4	49.6	0.8	8.7	27.5	18.4	32.5	47.7
		(38.2–48.2)	(27.5–36.5)	(25.0–34.2)	(44.8–54.5)	(0.3–2.3)	(6.0–11.4)	(23.3–32.2)	(14.7–22.1)	(28.0–37.4)	(42.9–52.5)
	Women	41.6	37.8	27.0	42.4	4.7	7.1	31.4	19.8	28.4	46.8
		(36.1–47.2)	(33.6–41.9)	(22.3–32.4)	(38.1–46.6)	(2.8–7.8)	(4.9–9.3)	(26.4–36.9)	(16.4–23.3)	(23.5–33.8)	(42.5–51.0)
60–69	Men	30.2	32.0	15.1	20.8	0	3.0	54.8	47.2	50.3	61.4
		(24.2–36.9)	(26.8–37.3)	(10.8–20.7)	(16.2–25.4)		(1.1–4.9)	(47.8–61.5)	(41.6–52.8)	(43.4–57.1)	(55.9–66.9)
	Women	23.2	30.6	7.4	22.1	0.5	4.4	69.5	47.4	47.8	59.1
		(17.9–29.4)	(25.7–35.5)	(4.5–11.8)	(17.7–26.5)	(0.1–2.7)	(2.2–6.6)	(62.8–75.4)	(42.0–52.7)	(41.0–54.6)	(53.9–64.3)
70–79	Men	32.8	30.0	15.8	13.5	0.6	0	51.5	56.5	63.7	75.9
		(26.2–40.1)	(23.1–36.9)	(11.1–22.0)	(8.4–18.7)	(0.1–3.2)		(44.0–58.8)	(49.0–63.9)	(56.3–70.6)	(69.5–82.3)
	Women	21.3	17.6	14.5	18.6	0	3.0	64.3	63.8	65.1	80.4
		(16.7–26.8)	(12.3–22.9)	(10.6–19.4)	(13.2–24.0)		(0.6–5.4)	(58.1–70.0)	(57.1–70.5)	(59.0–70.7)	(74.9–85.9)
80+	Men	20.9	21.6	20.9	21.6	0	0	58.1	56.8	79.1	89.2
		(11.4–35.2)	(8.4–34.9)	(11.4–35.2)	(8.4–34.9)			(43.3–71.6)	(40.8–72.7)	(64.8–88.6)	(79.2–99.2)
	Women	25.0	14.5	14.5	24.6	0	4.3	60.5	60.9	75.0	91.3
		(16.6–35.8)	(6.2–22.8)	(8.3–24.1)	(14.5–34.8)		(0–9.2)	(49.3–70.8)	(49.4–72.4)	(64.2–83.4)	(84.7–98.0)

Age is well known to be closely associated with refractive status. Myopia is generally more common in younger adults and hyperopia in older adults. The prevalence of myopia, however, shows a bimodal pattern in adult populations, initially decreasing with age and then increasing in the older populations [14–16, 21]. In the current study, the same bimodal pattern was found in the changes in the SE, but not in the spherical keratometric means. Moreover, myopic changes in the older age group (> 80 years old) were found only in eyes with significant cataract but not in those without cataract (Table 4). Thus, the current results support the theory that the collaboration of a hyperopic shift with ageing and myopic changes in the refractive index of the lens due to cataract formation should produce the bimodal pattern of refractive status [21, 34, 40, 41].

Hyperopic eyes increased with age in the current study and previous population-based studies [15, 17, 20, 42], which could be attributable to decreased refractive power of the lens [34, 40], changes in the lens position [15], flattened corneal curvature [43], and shortened axial length [43]. Since significant changes in corneal curvature with age was not found in the current study, other factors such as the lens power, lens position, or axial length should be more important for the hyperopic shift with age. The axial length showed negative correlation with age in the current study. Refractive astigmatism was positively correlated with age in this study population, which was consistent with previous studies [14, 17, 20, 23, 24, 34, 42, 44]. On the other hand, keratometric astigmatism was not significantly associated with age. In the vector analysis, the difference between refractive and keratometric astigmatism increased with increasing age. These findings suggest that an increase in refractive astigmatism is mainly due to changes in lens shape but not to changes in the cornea; lenticular astigmatism partially neutralized the effects of keratometric astigmatism in the younger age group.

The axis of astigmatism is known to be associated with age. Against-the-rule refractive astigmatism was common in the older age groups in the current study, which is consistent with previous studies [24, 42, 43]. Moreover, the polar value analyses in this study showed a trend toward against-the-rule astigmatism with increasing age in both refractive and keratometric astigmatism. Keratometric astigmatism was not correlated with age, whereas the polar value on keratometry decreased with increasing age, suggesting that the rotation of the astigmatic axis toward the against-the-rule form should be achieved without substantial changes in the magnitude of the keratometric astigmatism itself. Goss [45] proposed that eyelid tension is responsible for with-the-rule refractive astigmatism because of flatter corneas in the horizontal meridian and steeper corneas in the vertical meridian, leading to an age-dependent decrease in with-the-rule astigmatism with less lid tension.

The associations of sex with refractive errors were reportedly different among different countries or populations. In Asia, myopia was more common or hyperopia was less common in men than in women in Singapore [21], Indonesia [20], Bangladesh [24], India [22], and in the current study; however, such differences were not observed in Taiwan [34]. In Caucasian or African-American populations, a similar trend was found in some countries [14, 16, 33, 42], but the opposite was reported in others [13, 15]. In the current study, women had shorter axial length.

In the current study, axial length was significantly correlated with SE, refractive astigmatism, spherical keratometric mean, keratometric astigmatism, and sex after adjusting for age. These findings suggest that more hyperopic SE in women than in men was related to shorter axial length; myopia found in eyes with long axis are due not only to enlargement of axial length but also to the changes in corneal curvature. The central corneal thickness was not correlated with SE, keratometric mean, and refractive/keratometric astigmatism, suggesting that central corneal thickness has little effect on the corneal curvature and refractive status.

Possible limitations of the current study are the following. First, nearly one third of the participants were excluded because of the presence of pterygium. Since corneal curvature can be flattened with the elongation of pterygium [46], if eyes with pterygia are included in the current analysis, the average value of SE will be larger and the prevalence of hyperopia will increase. Second, the environmental or social factors, such as education [21, 22, 34, 47], income [20, 21], and housing [21], which can affect refractive status, could not be investigated in the current study because of the municipal laws for protecting the privacy of patient data. However, it can be speculated that the lower income of the islanders, which is almost half of that in mainland Japan [48], may have some association with the lower prevalence of myopia in the current study. Third, since accommodation can be found in up to approximately 50–55 years of age [49], the prevalence of myopia could be overestimated especially in younger age groups without the use of a cycloplegic agent and it should be better to use cycloplegic agent in epidemiological studies on refractive errors like The Teheran Eye Study [50]. However, because obtaining informed consent from all participants to determine cycloplegic refraction would have been very difficult, it was abandoned in the current study. Finally, the presence of selection bias should be discussed. In general, accurate determination of refractive status becomes more difficult in older individuals. In this study, the excluded participants were significantly older than the included participants. Since older individuals had more hyperopic refraction, the true prevalence of hyperopia would be higher than the current results.

In summary, in the rural population in a southern island of Japan, the prevalence of myopia is relatively higher than that in most other countries. The prevalence of high myopia was similar to that reported in Asian countries, but lower than that in Hispanic or white populations. However, these values were apparently lower compared to those in an urban city in the Japanese mainland, which may be due to environmental and genetic factors. The present data on refractive errors should play a vital role for planning effective eye care services in Japan, including rural areas, to reduce the visual morbidity due to refractive errors.
